# Comparative Analysis of Transperineal Cognitive Fusion, Systematic, and Combined Biopsies for Prostate Cancer Detection

**DOI:** 10.3390/medicina61122185

**Published:** 2025-12-09

**Authors:** Mihai Alexandru Radu, Sorin Cecil Mirea, Andrei Drocaș, Dragoș Vasile Florin, Nicoleta Alice Drăgoescu, George Mitroi, Andrei Pănuș, Petru Octavian Drăgoescu

**Affiliations:** 1 Department of Urology, University of Medicine and Pharmacy of Craiova, 200349 Craiova, Romania; 28radu.mihai@gmail.com (M.A.R.); andrei.drocas@umfcv.ro (A.D.); vdl_dragos@yahoo.com (D.V.F.); gmitroi@yahoo.com (G.M.); andrei.panus2@gmail.com (A.P.); pdragoescu@yahoo.com (P.O.D.); 2 Department of Surgery, University of Medicine and Pharmacy of Craiova, 200349 Craiova, Romania; 3 Department of Anaesthesiology and Intensive Care, University of Medicine and Pharmacy of Craiova, 200349 Craiova, Romania; alice.dragoescu@umfcv.ro

**Keywords:** prostate cancer, transperineal biopsy, cognitive targeting, systematic biopsy, multiparametric MRI, PI-RADS, clinically significant prostate cancer, ISUP grade

## Abstract

*Background and Objectives*: Multiparametric MRI (mpMRI) has improved prostate cancer (PCa) detection, but the added value of cognitive fusion (CF) over systematic biopsy (SB) remains debated. This prospective study evaluated the diagnostic performance of SB, CF, and their combined use in patients with Prostate Imaging and Reporting Data System (PI-RADS) ≥3 lesions. *Materials and Methods*: A total of 282 patients underwent mpMRI followed by both SB and CF biopsy. *Results*: PCa was diagnosed in 154 patients. SB detected 112 cancers (24 ISUP 1 (International Society of Urological Pathology), 88 ISUP ≥ 2), and CF detected 135 cancers (16 ISUP 1, 119 ISUP ≥ 2), while the combined approach detected all 154 cancers (9 ISUP 1, 145 ISUP ≥ 2). CF identified 42 cancers missed by SB, whereas SB identified 19 cancers not detected by CF. CF upgraded 38 patients from low-risk to intermediate-risk (23)/high-risk (15) categories, while SB underestimated disease severity in 41 cases. No major biopsy-related complications were recorded. *Conclusions*: CF biopsy outperformed SB in detecting clinically significant PCa and improved risk stratification, while the combined approach provided the highest overall diagnostic performance, supporting its use in contemporary PCa assessment.

## 1. Introduction

Prostate cancer is the second most commonly diagnosed non-skin cancer and the 6th leading cause of cancer-related death among male patients [1]. Studies and analyses confirmed wide geographic variations, with the highest incidence in the high-income regions and the highest mortality in the Caribbean and sub-Saharan Africa, while the rates have stabilized or declined in many developed countries [2]. Prostate-specific antigen (PSA) testing practices and evolving screening recommendations are strongly linked to these trends [3].

The 2025 EAU prostate cancer guidelines confirm that clinical suspicion is primarily based on the findings of digital rectal examination (DRE) and/or elevated PSA levels [4].

Diagnostic accuracy depends on the prostate sampling strategy. Since the introduction of random SB by Hodge et al., multiple extended biopsy protocols have been developed to improve accuracy [5]. Transperineal (TP) prostate biopsy has emerged as a valuable tool in prostate cancer diagnostics. Recent studies prove that, compared to transrectal approach, TP prostate biopsy provides at least comparable but in many cases superior detection rates [6,7]. Moreover, avoiding the rectal mucosa, TP prostate biopsy reduces infectious complications and also provides better access to anterior and apical prostate regions [8].

The introduction of mpMRI represents a significant advancement in prostate cancer diagnostics, enhancing tumour detection and localization. PI-RADS 2.1 [9] has improved diagnostic accuracy and consistency by enabling better targeting and reducing unnecessary procedures when used for fusion biopsy. Using mpMRI before or during biopsy has allowed for a more accurate diagnostic pathway [10,11,12].

Evidence from systematic reviews and randomized controlled trials challenges the traditional diagnostic method of performing a systematic 10–12 core transrectal ultrasound (TRUS)-guided biopsy in men with elevated prostate-specific antigen (PSA) levels [13,14,15,16].

However, when balancing cost-effectiveness with improved detection of significant cancers, MRI-targeted biopsy is still considerably more expensive than traditional methods. For example, in the U.S., the cost of an MRI-targeted biopsy is estimated at about $4000 per procedure, compared to roughly $2000 for a standard 12-core TRUS biopsy [17,18,19,20].

CF biopsy via the TP approach represents a valuable cost-effective alternative to software-based systems, mostly in low-resource settings, where access to advanced fusion technology is limited [19]. This technique enables accurate targeting of prostate cancer lesions without the need for expensive equipment, making MRI-guided biopsy more accessible in less developed healthcare systems.

The present study builds upon our previous work on the feasibility of TP cognitive-targeted biopsy [20] and addresses a central clinical question: whether cognitive targeting improves the detection of clinically significant prostate cancer compared with SB, and whether combining both techniques provides additional diagnostic value. Therefore, we aimed to (1) compare overall and clinically significant cancer detection rates across the three biopsy strategies; (2) quantify cancers missed by each method; (3) assess their impact on risk stratification; and (4) evaluate the safety profile of the TP approach.

## 2. Materials and Methods

### 2.1. Study Design

We conducted a prospective, single-centre observational study between October 2023 and June 2025 at the Department of Urology, County Emergency Clinical Hospital of Craiova, Romania. All procedures were performed within the urology unit of this institution. Written informed consent was obtained from all participants prior to enrolment. The study protocol was approved by the Institutional Ethics Committee of the University of Medicine and Pharmacy of Craiova (approval no. 203/20.09.2023) and adhered to the principles of the Declaration of Helsinki. Each patient underwent both systematic TP biopsy and CF-targeted biopsy, allowing within-patient comparison of the diagnostic performance of the three biopsy strategies.

### 2.2. Study Population

This study included 282 consecutive biopsy-naive patients evaluated for suspected prostate cancer. Baseline demographic and clinical characteristics such as age, PSA level, PSA density, prostate volume, DRE findings, PI-RADS score, and relevant comorbidities were collected for the entire cohort and are summarized in Table 1.

#### 2.2.1. Inclusion Criteria

Eligible patients were biopsy-naive adult men (≥18 years) who presented with clinical suspicion of prostate cancer, defined by elevated serum PSA levels above 4 ng/mL and/or an abnormal DRE. All individuals had undergone pre-biopsy mpMRI, and inclusion required the presence of at least one lesion scored PI-RADS 3, 4, or 5 according to PI-RADS v2.1. Only patients able and willing to provide written informed consent were enrolled.

#### 2.2.2. Exclusion Criteria

A total of 316 men were screened. Patients were excluded if mpMRI revealed no suspicious lesions (PI-RADS ≤ 2), if imaging data were incomplete or non-diagnostic, or if they declined participation. Additional exclusion criteria included a history of previous prostate biopsy or prostate-directed therapy, ongoing urinary tract infection or acute prostatitis, and any anatomical or medical condition that impeded safe TP access. Patients unable to tolerate the lithotomy position or the procedural requirements were also excluded.

A total of 316 individuals were initially screened; after applying these criteria, 282 patients were ultimately included in the analysis.

### 2.3. MRI Protocol and PI-RADS Assessment

mpMRI examinations were performed at the UMF Craiova Imaging Centre using a 3-Tesla Philips Ingenia scanner (Philips Healthcare, Amsterdam, The Netherlands). The mpMRI protocol included high-resolution T2-weighted sequences in axial, sagittal, and coronal planes, diffusion-weighted imaging with corresponding ADC maps, and dynamic contrast-enhanced sequences, in accordance with current PI-RADS v2.1 recommendations. All MRI studies were interpreted by experienced radiologists, and lesions were scored according to PI-RADS v2.1. Lesion location was documented using the standardized prostate sector map.

### 2.4. Biopsy Technique

All biopsies were performed transperineally under real-time ultrasound guidance using a Toshiba/Canon Aplio 500 system equipped with a biplanar probe (PVL-715RST) (Canon Medical Systems, Otawara, Japan). The procedure was conducted with the patient in the lithotomy position and under general anaesthesia, spinal (loco-regional) anaesthesia, or local anaesthesia, selected according to individual clinical characteristics, anaesthesiology evaluation, and patient preference. The majority of procedures in this cohort were performed under spinal anaesthesia, reflecting routine institutional practice. No antibiotic prophylaxis was administered, in line with current evidence supporting the very low infectious risk associated with the TP route and contemporary antibiotic stewardship principles.

**Figure 1:**
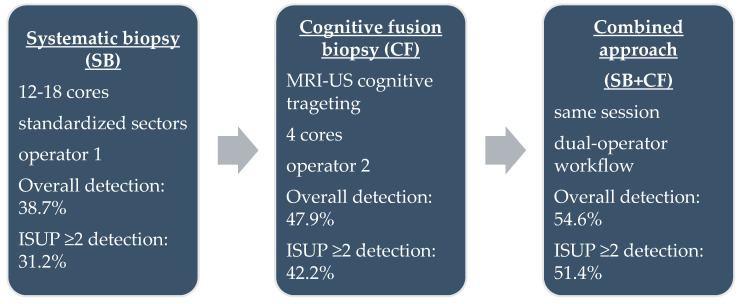
Schematic representation of the three biopsy protocols.

Each patient underwent both SB and CF during the same session (Figure 1). Systematic sampling was performed first by a dedicated operator, who obtained 12–18 cores according to the Vienna nomogram [21], targeting standardized sectors of the peripheral and transitional zones. CF biopsy was subsequently performed by a second operator, who mentally correlated mpMRI-identified lesions with real-time ultrasound anatomy. For each PI-RADS ≥ 3 lesion, four targeted cores were obtained. All biopsies were performed using single-use 18 G × 25 cm Bard needles and a Max-Core™ spring-loaded biopsy device (Bard Peripheral Vascular, Inc., Tempe, AZ, USA). This dual-operator workflow was designed to minimize cognitive bias, ensure procedural consistency, and allow independent assessment of each biopsy approach.

### 2.5. Outcomes

The primary outcome of the study was the detection rate of clinically significant prostate cancer (csPCa), defined as ISUP grade group ≥2. Secondary outcomes included the overall prostate cancer detection rate, the number of csPCa cases missed by each biopsy approach when considered independently, and the detection of high-grade prostate cancer (ISUP grade groups 4–5). Additional secondary measures included changes in D’Amico risk [22] classification derived from each biopsy method and the periprocedural complication rate, which was recorded and graded according to the Clavien–Dindo classification system.

### 2.6. Statistical Analysis

Continuous variables were assessed for normality using the Kolmogorov–Smirnov test and reported as mean with standard deviation or median with interquartile range, as appropriate. Categorical variables were expressed as frequencies and percentages. Prostate cancer detection rates for each biopsy method were calculated as proportions with corresponding 95% confidence intervals. Comparisons between biopsy techniques (systematic, CF, and combined) were performed using the chi-square test or Fisher’s exact test, with Bonferroni adjustment applied for multiple pairwise comparisons. Risk ratios with 95% confidence intervals were calculated to evaluate relative differences in csPCa detection.

A post hoc power analysis was conducted to assess whether the study sample size was sufficient to detect differences in csPCa detection rates between biopsy methods. Statistical analyses were performed using MedCalc Statistical Software version 20.218 (MedCalc Software Ltd., Ostend, Belgium). A two-tailed *p*-value below 0.05 was considered statistically significant.

## 3. Results

### 3.1. Overall Cancer Detection

A total of 154 patients were diagnosed with prostate cancer using the combined SB and CF biopsy approach, corresponding to a detection rate of 54.6% (95% CI 48.8–60.3). Among these, 9 tumours were classified as ISUP grade group 1 and 145 as ISUP grade group ≥2 (Table 2).

### 3.2. Detection by Biopsy Technique

**Figure 2:**
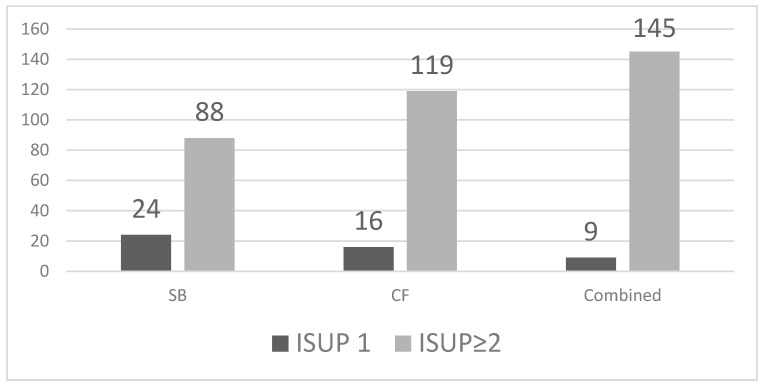
Detection of ISUP 1 and ISUP ≥2 prostate cancer by biopsy method.

SB detected 112 cancers (39.7%, 95% CI 34.2–45.5), including 24 ISUP 1 and 88 ISUP ≥ 2 tumours. CF biopsy identified 135 cancers (47.9%, 95% CI 42.1–53.7), of which 16 were ISUP 1 and 119 were ISUP ≥ 2. The combined approach detected all 154 cancers in our cohort (54.6%, 95% CI 48.8–60.3), with 9 ISUP 1 and 145 ISUP ≥ 2 tumours. CF outperformed SB in overall and clinically significant cancer detection (Figure 2).

### 3.3. Overlap Between Biopsy Techniques

**Figure 3:**
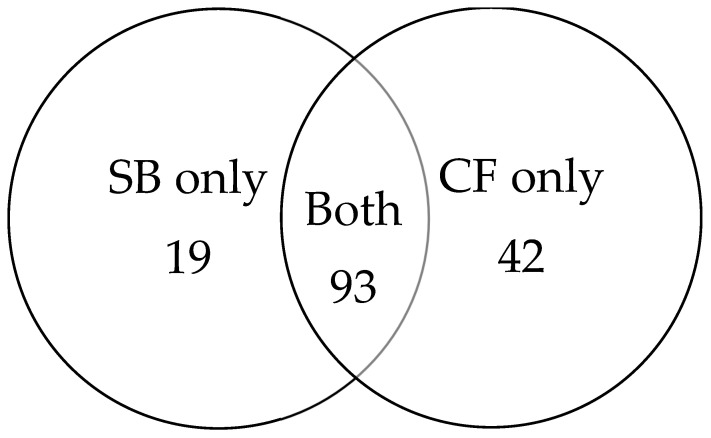
Venn diagram illustrating cancer detection overlap between SB and CF.

Among the 154 cancer cases, 93 were detected by both biopsy methods. An additional 19 cancers were identified exclusively by SB, while 42 cancers were detected only by CF (Figure 3). CF provided an incremental gain of 27.3% over SB.

### 3.4. Clinically Significant Prostate Cancer

Clinically significant prostate cancer (ISUP ≥ 2) was detected in 88 patients with SB (31.2%, 95% CI 26.1–36.8), in 119 patients with CF (42.2%, 95% CI 36.6–48.0), and in 145 patients with the combined approach (51.4%, 95% CI 45.6–57.2). CF identified 31 additional ISUP ≥2 cancers compared with SB, while the combined technique identified 57 more clinically significant tumours than SB alone. Compared with SB, CF increased the likelihood of detecting clinically significant prostate cancer (RR 1.35, 95% CI 1.08–1.69), and the combined approach further improved detection (RR 1.65, 95% CI 1.34–2.03). The difference in detection between CF and the combined approach reached statistical significance (*p* = 0.04). SB missed 31 clinically significant tumours (ISUP ≥ 2) that were detected only by CF, whereas CF missed 14 clinically significant tumours identified exclusively by systematic sampling (Table 3).

### 3.5. Risk Reclassification

CF biopsy upgraded 38 patients from low-risk to intermediate- or high-risk disease according to the D’Amico classification. SB underestimated disease severity compared with CF in 41 patients (Table 4).

### 3.6. PI-RADS Categories

Cancer detection increased with PI-RADS category, with the highest rates observed in PI-RADS 4 and PI-RADS 5 lesions. CF consistently demonstrated higher detection rates than SB across all PI-RADS categories, and the combined approach had the highest proportion of detected cancers.

### 3.7. Lesion-Level Performance

CF biopsy demonstrated superior lesion-level sensitivity, particularly for lesions showing restricted diffusion or larger dimensions. SB contributed to the detection of a limited number of cancers in lesions with low conspicuity on MRI, supporting its complementary role. The combined strategy ensured full sampling of all MRI-visible lesions.

### 3.8. PSA and PSA Density

Higher PSA density was associated with an increased likelihood of clinically significant disease. Patients with PSA density ≥ 0.15 ng/mL/mL showed a higher proportion of ISUP ≥ 2 tumours. CF maintained superior detection performance across PSA density subgroups, and the combined approach achieved the highest detection rates overall.

### 3.9. Procedure-Related Complications

Procedural complications were generally mild. Transient lower urinary tract symptoms (LUTS) occurred in 18 patients (6.4%), of whom 6 were diagnosed with urinary tract infection based on clinical and laboratory findings and were successfully treated with oral antibiotics. Macroscopic haematuria was observed in 14 patients (5%), with spontaneous resolution in all cases. Perineal discomfort was reported by 22 patients (7.8%). No episodes of fever, urinary retention requiring prolonged catheterization, or sepsis were recorded, and no Clavien–Dindo grade ≥ 2 complications occurred. Overall, the TP biopsy approach demonstrated a favourable safety profile (Table 5).

## 4. Discussion

Cognitive MRI–ultrasound fusion biopsy proved to be a practical and effective alternative to software-guided fusion in our study, particularly in a resource-limited setting. Careful mpMRI review in collaboration with an experienced radiologist, together with a biplane TP ultrasound approach, enabled accurate cognitive alignment and reliable targeting of suspicious lesions. These observations align with recent evidence reinforcing the strong correlation between mpMRI-identified lesions and final histopathology [23], as well as the high diagnostic performance of mpMRI-guided strategies in both initial and repeat biopsy settings [24]. In addition, the CF technique offers the advantage of substantially lower cost and wider accessibility compared with software-based fusion systems, as it does not require dedicated hardware or additional imaging equipment. Previous cost analyses have shown that CF reduces procedural expenses by approximately 40–60% while maintaining comparable detection rates [6]. In Romania, this difference is particularly relevant: in private clinics, the out-of-pocket cost for patients is approximately 600 USD for software-assisted fusion biopsy, whereas standard TP or cognitively targeted biopsy is around 375 USD. Moreover, software-based fusion platforms are generally unavailable in the public healthcare sector, where only cognitive targeting can be implemented in daily practice. This reinforces the practical and economic importance of CF as a cost-efficient and accessible strategy, especially in resource-limited settings.

Our results confirm that combining systematic and cognitive-targeted biopsies provides superior diagnostic performance compared with either method alone. In our cohort of 282 patients, the overall prostate cancer detection rate was 54.6% for the combined approach, compared with 39.7% for SB and 47.9% for CF. Although CF outperformed systematic sampling in overall cancer detection, the difference between the two techniques remained moderate, a finding consistent with previous studies assessing these strategies independently. Importantly, each method compensated for the limitations of the other, with CF improving detection in regions typically undersampled by SB, and vice versa. Several of these uniquely detected cases represented clinically significant tumours, underscoring the complementary diagnostic value of the two approaches. These observations parallel findings from larger studies, including the PAIREDCAP trial, which reported that 11–33% of clinically significant cancers would be missed if only one biopsy modality were used, and previous reports, where the combined approach detected approximately 10% more clinically relevant cancers than either method alone [25,26]. Collectively, these results support the integration of systematic and targeted sampling to maximize diagnostic sensitivity in contemporary TP biopsy practice.

The addition of cognitive-targeted cores resulted in a clear improvement in the detection of clinically significant prostate cancer at the cohort level. When expressed as a proportion of all 282 biopsied patients, ISUP grade ≥ 2 tumours were detected in 31.2% (95% CI 26.1–36.8) of men by SB alone (88/282), in 42.2% (95% CI 36.6–48.0) by CF alone (119/282), and in 51.4% (95% CI 45.6–57.2) when both techniques were combined (145/282). This stepwise increase highlights the incremental diagnostic contribution of targeted sampling, which is particularly relevant for identifying higher-grade lesions. In line with prior evidence, MRI-guided targeting preferentially identifies aggressive cancers while reducing the detection of indolent disease. The meta-analysis by Wegelin et al. reported that MRI-targeted biopsy detects significantly more clinically significant cancers and fewer low-grade tumours than SB, with no clear performance difference between cognitive, software-based, or in-bore targeting modalities [27]. Our findings are consistent with these observations: CF increased the detection rate of ISUP ≥ 2 cancers while contributing relatively fewer ISUP 1 diagnoses, supporting the concept that MRI-informed biopsy pathways enhance the focus on clinically relevant disease and help limit unnecessary overtreatment.

The use of TP CF in our workflow also translated into more accurate risk stratification. CF upgraded 38 patients from low-risk to intermediate- or high-risk categories according to the D’Amico classification, while SB underestimated disease severity in 41 patients. These findings indicate that relying on SB alone would have resulted in substantial underestimation of tumour aggressiveness. This reclassification is clinically meaningful because ISUP 1 disease is typically managed with active surveillance, whereas ISUP ≥ 2 usually warrants curative treatment. Therefore, detecting additional intermediate- and high-risk cancers through CF or the combined approach reduces the risk of undertreatment, while better discrimination of true ISUP 1 cases helps avoid unnecessary interventions. Although treatment decisions and follow-up strategies were not analyzed in this study, the observed risk reclassification suggests that combined biopsy could influence downstream management, particularly by shifting patients from active surveillance to definitive therapy. Future studies with longitudinal follow-up are needed to determine how biopsy-driven reclassification translates into treatment selection and clinical outcomes. Our observations align with prior work by Ahdoot et al., who reported grade-group upgrading in more than 20% of patients when targeted cores were integrated with systematic sampling [26]. Overall, the incorporation of CF improved classification accuracy and ensured that patients with higher-risk disease were more reliably identified and appropriately managed.

Earlier studies of CF have shown mixed results, often influenced by biopsy route and operator experience. Delongchamps et al. [28] reported no clear advantage of visually directed biopsies over systematic sampling in a transrectal setting, whereas Pepe et al. [29] demonstrated that CF markedly improves the detection of significant cancers, particularly by reducing the risk of missing anterior lesions. Similarly, CF improved sampling of anterior and apical lesions, which are commonly missed by SB. These data highlight the importance of both biopsy route and operator expertise. When performed by experienced teams with meticulous MRI–ultrasound alignment, CF can approach the diagnostic performance of software-based fusion systems. Nevertheless, because accuracy may be lower for small or subtly defined targets, appropriate training remains essential, and software fusion may retain value in selected cases, despite its higher cost and limited accessibility. To ensure reproducibility across centers, CF requires structured training in mpMRI interpretation and standardized targeting workflows. Multicentre studies evaluating learning curves and quality-assurance protocols would help clarify how reliably this technique can be adopted in routine clinical practice [28,30].

Recent data from Olivetta et al. [29] further support the clinical relevance of cognitively targeted biopsy, although within a different diagnostic framework. In their single-centre retrospective analysis, cognitive targeting alone was evaluated as a standalone technique in biopsy-naïve men with PI-RADS ≥ 3 lesions, having an overall cancer detection rate of 50% and a csPCa detection rate of 33%. Unlike our paired-design study, their protocol did not include systematic sampling, which likely explains the lower csPCa detection rates compared with our combined approach. Nonetheless, their findings confirm that CF performs well in identifying MRI-visible disease and can function as a feasible pathway in selected patients. When placed in context, our results highlight that while CF alone can detect a substantial proportion of significant cancers, the combined TP protocol continues to provide superior diagnostic accuracy, particularly by reducing missed high-grade tumours. These differences underscore the importance of biopsy strategy selection and support the broader applicability of CF across various institutional settings.

Recent multicentre evidence also supports the diagnostic utility of transperineal CF. Hung et al. [31] analyzed 490 men undergoing TP MRI-targeted biopsy and reported comparable detection of clinically significant prostate cancer between cognitive and software fusion, both in unadjusted analyses (51% vs. 39%) and after multivariable adjustment (OR 1.46; 95% CI 0.82–2.58). CF achieved similar performance despite requiring fewer systematic cores and no dedicated fusion hardware, indicating its feasibility in routine practice and in centers with limited technological resources. These results are consistent with our findings and further support CF as an efficient and accessible MRI-directed biopsy strategy.

Beyond overall detection, the clinically relevant question is how many significant cancers each biopsy method fails to identify. In our cohort, SB missed 31 clinically significant tumours (ISUP ≥ 2) that were detected only by CF, whereas CF missed 14 clinically significant tumours identified exclusively by systematic sampling. When considering all cancers, systematic sampling missed 42 cases and cognitive targeting missed 19, confirming that neither technique alone ensures complete diagnostic coverage. Only the combined approach eliminated missed high-grade disease, underscoring the complementary nature of the two strategies and reinforcing the rationale for an integrated biopsy protocol in biopsy-naïve patients.

Clinically, CF alone may be sufficient in patients with clearly defined PI-RADS 4–5 anterior or apical lesions, where systematic sampling adds limited incremental value. In contrast, the combined approach remains essential in multifocal disease, heterogeneous MRI conspicuity, or PI-RADS 3 lesions, where relying solely on targeted sampling risks missing significant tumours. CF also reduced the total number of targeted cores per lesion, without compromising diagnostic performance, a feature that may streamline workflow and reduce tissue burden.

This study has several limitations. It was conducted in a single centre with a moderate sample size, which may limit generalizability, and although all procedures were performed by experienced operators, some degree of inter-operator variability cannot be excluded. We did not evaluate a parallel arm using software-assisted or in-bore MRI fusion, and therefore our comparisons are limited to the cognitive and systematic approaches used within our workflow. In addition, lesion location was not systematically recorded, preventing a more detailed analysis of whether anterior or apical regions accounted for most cognitively detected cases. Long-term oncological outcomes were also not available, and we did not perform a formal assessment of cost-effectiveness, which may be relevant when selecting biopsy strategies in different resource environments. Larger multicentre studies with standardized reporting and extended follow-up are needed to validate these findings and further define the role of TP CF in contemporary prostate cancer diagnostics.

## 5. Conclusions

The combined use of systematic and cognitive-targeted TP biopsy provided the highest overall and clinically significant prostate cancer detection rates in our cohort. Neither technique alone was sufficient, as each missed relevant tumours that the other identified. CF improved sampling of MRI-suspicious regions, while systematic cores remained essential for detecting lesions outside targeted areas. These findings reinforce the value of an integrated biopsy strategy in contemporary clinical practice and support the continued use of both approaches to maximize diagnostic sensitivity.

## Figures and Tables

**Table 1:** Demographic and clinical characteristics of the study population (*n* = 282).

Variable	Value (*n* = 282)
Age (years)	72 (IQR 66–77)
Weight (kg)	79.9 ± 12.7
BMI	26.5 ± 3.9
Prostate volume (cc)	48 (IQR 37–61)
PSA (ng/mL)	9.8 (IQR 6.5–13.2)
PSA density (ng/mL/cc)	0.21 (IQR 0.13–0.29)

**Table 2:** Cancer detection according to biopsy technique.

Method	ISUP 1	ISUP ≥ 2	Total PCa
SB	24	88	112
CF	16	119	135
Combined	9	145	154

**Table 3:** Distribution of prostate cancer cases by ISUP grade and Gleason score, comparing CF, SB, and combined biopsy approaches. CF detected slightly more clinically significant cancers (ISUP ≥ 2) than SB, but with no statistical significance (*p* = 0.06).

ISUP Score	Gleason Score	CF (*n* = 135)	SB (*n* = 112)	Combined (*n* = 154)	CF vs. SB	*p*-Value
1	3 + 3	16	24	9	–8	0.09
2	3 + 4	35	28	45	7	0.38
3	4 + 3	24	20	28	4	0.81
4	4 + 4, 3 + 5	24	18	30	6	0.54
5	4 + 5, 5 + 4, 5 + 5	36	22	42	14	0.17
≥2	≥7	119	88	145	31	0.06

**Table 4:** Upgrade and underestimation rates when comparing SB with CF according to ISUP grade groups.

Parameter	Number of Patients
Upgraded by CF (From ISUP 0–1 to ISUP ≥ 2)	38
Underestimated by SB (CF higher grade than SB)	41

**Table 5:** Procedural Complications.

Complication Type	*n* (%)	Notes
LUTS	18(6.4%)	6/18 had confirmed UTI (Urinary Tract Infection) and required antibiotics
Urinary tract infection	6 (2.1%)	All cases treated successfully with antibiotics; no hospitalizations
Macroscopic haematuria	14 (5.0%)	Resolved spontaneously in all cases
Perineal discomfort	22 (7.8%)	Mild, self-limited

## Data Availability

The data presented in this study are available on request from the corresponding author.

## Abbreviations

The following abbreviations are used in this manuscript:

PCa	Prostate Cancer
PSA	Prostate-Specific Antigen
DRE	Digital Rectal Examination
mpMRI	Multiparametric Magnetic Resonance Imaging
PI-RADS	Prostate Imaging and Reporting Data System
TP	Transperineal
CF	Cognitive Fusion
SB	Systematic biopsy
ISUP	International Society of Urological Pathology
csPCa	Clinically significant prostate cancer
IQR	Interquartile Range
RR	Risk Ratio
UTI	Urinary tract infection
LUTS	Lower Urinary Tract Symptoms
TRUS	Transrectal ultrasound
